# Adult perioperative cardiac arrest: An overview of 684 cases reported to webAIRS

**DOI:** 10.1177/0310057X231196912

**Published:** 2023-10-06

**Authors:** Matthew R Bright, Yasmin Endlich, Zachary DJ King, Leigh D White, Sandra I Concha Blamey, Martin D Culwick

**Affiliations:** 1 Department of Anaesthesia, Princess Alexandra Hospital, Woolloongabba, Australia; 2 Faculty of Medicine, University of Queensland, St. Lucia, Australia; 3 Department of Anaesthesia, 1062 Royal Adelaide Hospital, Adelaide, Australia; 4 Faculty of Medicine, The University of Adelaide, Adelaide, Australia; 5 Australian and New Zealand Tripartite Anaesthesia Data Committee, Melbourne, Australia; 6 Department of Anaesthesia, Royal Brisbane & Women’s Hospital, Herston, Australia; 7 Department of Anaesthesia and Perioperative Medicine, Sunshine Coast Hospital and Health Service, Birtinya, Australia

**Keywords:** Perioperative, cardiac arrest, mortality, complications

## Abstract

There were 684 perioperative cardiac arrests reported to webAIRS between September 2009 and March 2022. The majority involved patients older than 60 years, classified as American Society of Anesthesiologists Physical Status 3 to 5, undergoing an emergency or major procedure. The most common precipitants included airway events, cardiovascular events, massive blood loss. medication issues, and sepsis. The highest mortality rate was 54% of the 46 cases in the miscellaneous category (this included 34 cases of severe sepsis, which had a mortality of 65%). This was followed by cardiovascular precipitants (*n* = 424) in which there were 147 deaths (35% mortality): these precipitants included blood loss (53%), embolism (61%) and myocardial infarction (70%). Airway and breathing events accounted for 25% and anaphylaxis 8%. A specialist anaesthetist attended the majority of these cardiac arrests. As webAIRS is a voluntary database, it is not possible to determine the incidence of perioperative cardiac arrest and only descriptive information on factors associated with cardiac arrest can be obtained. Nevertheless, the large number of reports includes a wide range of cases, precipitants, demographics and outcomes, providing ample opportunity to learn from these events. The data also provide rich scope for further research into further initiatives to prevent cardiac arrest in the perioperative period, and to improve outcomes, should a cardiac arrest occur.

## Introduction

Globally, 310 million surgeries are performed yearly, the majority most likely requiring anaesthetic perioperative care.^1^ Fortunately, anaesthesia has become very safe and perioperative cardiac arrests are rare.^1–4^ Nevertheless, when they occur, they have a high mortality risk, may cause significant trauma and harm to the patient, and might considerably affect the treating anaesthetist and the perioperative team. Cardiac arrest associated with anaesthesia is defined as the sudden loss of cardiac function resulting in circulatory failure from the time the patient is checked in for an operation until 24 h postoperatively.^5^ The last Australian analysis of cardiac arrests in the perioperative period was published in 1993. It identified 87 cases reported to the Australian Incident Monitoring Study (AIMS).^4^ Most incidents were related to drug administration (opioid, volatile anaesthetic agent, muscle relaxant or beta blocker). Other common factors included hypoventilation and abnormal surgical bleeding.^4^ An anaesthetic cause was identified in 46% of cases, and 58% of cases were deemed preventable by the reporters.^4^ Several clinical recommendations followed this analysis, many of which are now considered a minimum perioperative standard of care.^4^

The webAIRS incident monitoring system started collecting voluntary de-identified reports of anaesthesia incidents in Australia and New Zealand in September 2009 and is owned, managed and funded by three organisations: the Australian and New Zealand College of Anaesthetists (ANZCA), the Australian Society of Anaesthetists and the New Zealand Society of Anaesthetists. All registered anaesthetists in Australia and New Zealand have been invited to report anaesthetic incidents via the webpage www.anztadc.net. Data from webAIRS reports and analyses are regularly presented at scientific meetings and in peer-reviewed professional journals and anaesthetic magazines.

By March 2022, 8618 reports involving adult patients had been received. This article aims to provide an overview of all incidents involving cardiac arrest reported to webAIRS over this time, to identify potential risk factors and characterise likely causes, key precipitants, and outcomes.

## Methods

Data were collected in compliance with the National Health and Medical Research Council 2014 recommendations for de-identified quality assurance data.^6^ To ensure that the project meets the requirements for de-identification, multicentre ethics approval is maintained by the Royal Brisbane and Women’s Hospital Human Research Ethics Committee (HREC/11/QRBW/311) and the Nepean Blue Mountains Local Health District (HREC/12/NEPEAN/18). In New Zealand, ethics approval has been obtained from the Health and Disability Ethics Committee (MEC/09/17/EXP). All ethics committees suggested that the data collected for age and duration of the procedure be grouped, rather than by actual age or duration of the procedure, to reduce the chance of surrogate identification. Data describing the incidents can be entered by the reporter via non-mandatory data input web controls, and in the form of text, allowing the reporters to describe the incident and enter their reflections regarding contributing and alleviating factors.

A narrative search using Structured Query Language (SQL) was performed upon reports submitted from webAIRS’ inception in September 2009 to 31 March 2022 to identify events that might have involved cardiac arrest in patients who were more than 17 years of age. The SQL search was designed to return reports coded as ‘cardiac arrest’ by reporters or reports containing the partial words ‘arrest, CPR, resus, death, die, fatal’ in the description of the incident in the narrative section.

Reports were included when they were coded as a ‘Cardiac Arrest’ via data input web controls or described as a ‘Cardiac Arrest’ by the reporter in the narrative. Reports were also included when one or more of the following events were described: ventricular tachycardia, ventricular fibrillation, asystole, or pulseless electrical activity. In addition, all events where cardiopulmonary resuscitation (CPR) was commenced were included, as well as shockable and non-shockable cardiac arrests.^5,7^ During the analysis, the search terms used returned some reports that described low cardiac output states and near cardiac arrest, but CPR was not performed, and direct current countershock was not applied. These cases were coded as peri-arrest and excluded from the analysis. Any reports of patients who died or were palliated within 24 h but did not receive chest compression or defibrillation were excluded.

Data were extracted from the webAIRS database for the analysis group, which included demographic data of the patient including age in years (age), biological sex (sex), American Society of Anesthesiologists Physical Status Classification (ASA PS), body mass index (BMI) kg/m^2^, surgical procedure information, urgency (elective or emergency), specialty, time of day (i.e. 08:00 to 18:00 h, or out of hours 18:00 to 07:59 h), hours since an 8-h break, as well as information on anaesthesia (anaesthetic technique and level of experience of anaesthetist). Where data were missing for the codified data (e.g. ASA PS, age, sex, or urgency of procedure), these data were added if mentioned in the narrative section; otherwise, they were described as ‘not specified’. The mortality rate for each parameter of each demographic group was determined. A further comparison was made within the parameters of each demographic group to compare the frequency of mortality of one parameter in the group, chosen as a divisor, with the other parameters. For instance, using ASA PS as an example of one of the demographic factors studied, the parameter ASA PS 1 was used as the divisor to compare with the mortality of ASA PS 2, 3, 4 and 5 to calculate the risk ratio within this series. This was repeated for all the demographic groups studied. Mortality for the purposes of this analysis was defined as those cases that were coded by the reporter as having died or were stated to have died in the narrative sections describing the cardiac arrest, excluding cases that were palliated.

The analysers classified the precipitating event prior to the cardiac arrest using the existing webAIRS main category and subcategory classifications. For all cases, the classifications were based on the most likely key event in the chain of events before cardiac arrest and were classified as the ‘key precipitant’. In cases where more than one event was possible, the authors jointly assigned, based on the provided information, the most probable key precipitant. A small number where this was not possible were coded as ‘Other’. The timing of cardiac arrest was based on the reporter’s definition from the webAIRS narrative report or matched to one of the following definitions: preoperative cardiac arrest was defined as occurring at any time before any anaesthetic drug was administered; cardiac arrest at induction was defined as occurring in the first 10 min after the administration of anaesthetic drugs; postoperative cardiac arrest was defined as occurring in the time from discontinuation of the administration of volatile or hypnotic drugs until 24 h later. Postoperative cardiac arrest was further divided into emergence, which was the initial postoperative period of awakening from anaesthesia until handover in the post-anaesthesia care unit (PACU). The period after discharge from the PACU was further divided into intensive care unit (ICU), high dependency unit (HDU) or ward, depending on the patient’s location for the remainder of the 24-h period.

Outcomes from cardiac arrest were categorised into whether CPR was performed, patient disposition (defined as transfer to ICU, HDU or hospital ward) or any in-hospital mortality (as defined above).

All cases of cardiac arrest were then classified based on contributing factors using slightly modified ANZCA mortality audit^8^ definitions into most likely attributable factors:

- Under the control of the anaesthetist or related to anaesthesia;
- Under the control of the surgeon or related to surgery;
- Combination of anaesthetic and surgical factors;
- Other than anaesthesia or surgery.

A minimum of two authors assessed and categorised information extracted from the narrative review. Any discrepancy that could not be resolved was discussed with a third author (YE) or brought to a case discussion with all authors to reach a consensus.

## Results

**Figure 1.**
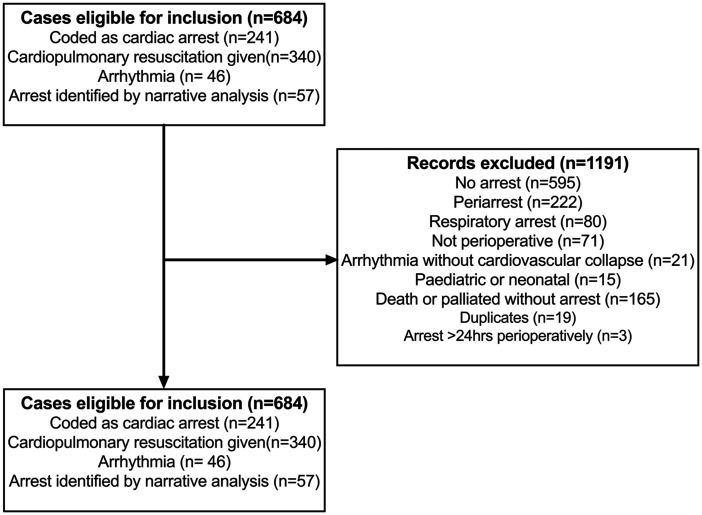
Flow diagram of webAIRS incident reports to identify cases of adult perioperative cardiac arrest.

During the data collection period there were 8618 incident reports in the webAIRS database involving adults. A total of 1875 matched the initial search criteria and were reviewed. Of these, 684 cases of perioperative cardiac arrest were eligible for inclusion. The remaining 1191 cases did not meet inclusion criteria and were not reviewed (Figure 1). These included patients who did not require CPR because they were considered peri-arrests and CPR was not required, or because CPR was considered futile by the treating team.

Data on peri-arrests were not analysed in detail, but the authors noted that many of these were triggered by vagal stimulation or were related to anaphylaxis. The former presented as severe bradycardia and either recovered spontaneously or after receiving anticholinergic drugs. Early administration of 50–200 µg of intravenous adrenaline appeared to be a common theme in cases of peri-arrest with refractory hypotension that did not progress to cardiac arrest.

Of the 684 cases reported, ASA PS 3 (34.4%) was the most common physical status classification, followed by ASA PS 4 (26.9%) and ASA PS 2 (26.8%) (Table 1). About 52% were reported as males and 45% as females (Table 1). A BMI greater than 30kg/m^2^ (*n* = 240, 35.1%) was more frequently reported than the other BMI groups, but only slightly more frequently than normal BMI (32%), and mortality was similar for all BMI groups (around 30%), except for the underweight group at 61% (Table 1). About 62% were older than 60 years and the mortality of this group was over 80%. Nearly half (46.1%) were emergency cases, of which about 28% were performed outside normal working hours. The mortality for after-hours cardiac arrest was around 45% for both the 18:00 h to 22:00 h period (43%) and for the 22:00 h to 07:59 h period (47%), compared with a mortality of 27% for the 08:00 h to 18:00 h period. Most of the arrests (82.5%) occurred when the anaesthetist had been on duty for 10 h or less. About 10% occurred when the anaesthetist had been on duty for more than 10 h, and in the remaining cases the hours on duty were not specified. Mortality was higher when the anaesthetist had been on duty for more than 10 h. The hours since an 8-h break were also analysed. In a similar pattern to hours on-duty, the majority of cardiac arrests occurred when it had been less than 10 h since the anaesthetist’s last 8-h break (72.4%). Almost 14% of the cardiac arrests occurred when the anaesthetist had been on duty for more than 10 h since the last 8-h break, with 2% occurring when it had been greater than 24 h since the last 8-h break. There was about 50% greater the mortality in the group where it was 16 to less than 24 h since the anaesthetist’s last 8-h break, but otherwise the mortality was similar to the group where the anaesthetist had less than 10 h since their last 8-h break. General anaesthesia was the most common group (82.2%) in the category of type of anaesthesia involving hypnotics, with an associated mortality of around 29%. There was a small number of cases (*n* = 16, 2.3%) where hypnotics were not involved but the mortality in this group was nearly 44%. Cases under sedation accounted for about 9% of cases, with a mortality of 39%. Cases involving regional anaesthetic techniques accounted for 18% of the arrests, with a mortality of 30%, and cases involving other local anaesthetic techniques accounted for 7.6% of arrests, with a mortality of 36.5%. It should be noted than some cases might have involved a combined general anaesthetic and regional technique. The most common location where cardiac arrest occurred was the operating theatre (*n* = 540, 79%), with a mortality of 28%. The numbers were fewer, but the mortality rate was higher for the cardiology lab (56%), gastroenterology department (30%), PACU (45%), radiology department (56%) and other (45%). Most of the cases were performed by a specialist anaesthetist (83%). The most common surgical procedures were orthopaedic (22%) and general surgery (22%).

**Table 1.** Demographics and mortality rates of 684 perioperative cardiac arrests identified from the webAIRS database.

	Number	Percent	Mortality (*n*)	Mortality %	RR (death)
ASA Physical Status Classification
1	37	5.4%	2	5.4%	Divisor
2	183	26.8%	15	8.2%	1.52
3	235	34.4%	62	26.4%	4.88
4	184	26.9%	92	50.0%	9.25
5	40	5.8%	32	80.0%	14.80
Not specified	5	0.7%	0		
Total	684		203		
Mortality % is the mortality compared with the number of arrests for the same row. For instance, ASA PS 1 had 37 arrests and two deaths, giving a mortality after cardiac arrest of 5.4%. It is not a percentage of the 206 deaths, which for ASA PS 1 would be about 1%. RR (death) is the relative ratio between the percent of the mortality for the row and the divisor (full explanation in Methods.)					
These abbreviations and methods also apply to all subsequent rows in Table 1.					
Biological sex
Female	308	45.0%	75	24.4%	Divisor
Male	354	51.8%	124	35.0%	1.44
Not specified	22	3.2%	4		
Total	684		203		
Age (years)
17–39	81	11.8%	4	4.9%	Divisor
40–59	168	24.6%	35	20.8%	4.22
60–79	281	41.1%	84	29.9%	6.05
80+	144	21.1%	79	54.9%	11.11
Not specified	10	1.5%	1		
Total	684		203		
Body mass index (kg/m^2^)
<18.5 (underweight)	18	2.6%	11	61.1%	2.12
18.5–25 (normal weight)	219	32.0%	63	28.8%	Divisor
25–30 (overweight)	119	17.4%	33	27.7%	0.96
Above 30 (obese)	240	35.1%	63	26.3%	0.91
Not specified	88	12.9%	33		
Total	684		203		
Emergency
Elective	359	52.5%	60	16.7%	Divisor
Emergency	315	46.1%	141	44.8%	2.68
Not specified	10	1.5%	2		
Total	684		203		
Time of day
08:00-18:00	554	81.0%	151	27.3%	Divisor
18:00-22:00	68	9.9%	29	42.6%	1.56
22:00-07:59	38	5.6%	18	47.4%	1.74
Not specified	24	3.5%	6		
Total	684		203		
Hours on duty
0 to <10 h	564	82.5%	155	27.5%	Divisor
10 to <16 h	54	7.9%	25	46.3%	1.68
16 to <24 h	9	1.3%	5	55.6%	2.02
More than 24 h	5	0.7%	1	20.0%	0.73
Not specified	52	7.6%	17		
Total	684		203		
Hours since 8-h break
0 to <10 h	495	72.4%	142	28.7%	Divisor
10 to <16 h	64	9.4%	20	31.3%	1.09
16 to <24 h	16	2.3%	7	43.8%	1.53
More than 24 h	14	2.0%	2	14.3%	0.50
Not specified	95	13.9%	32		
Total	684		203		
Anaesthesia and sedation
General anaesthesia	562	82.2%	160	28.5%	Divisor
Sedation	62	9.1%	24	38.7%	1.36
Hypnotics not involved	16	2.3%	7	43.8%	1.54
Not specified	30	4.4%	6	20.0%	0.70
Other	14	2.0%	6	42.9%	1.51
Total	684		203		
Regional and local techniques
Regional anaesthesia	123	18.0%	37	30.1%	Divisor
Local anaesthetic (other)	52	7.6%	19	36.5%	1.21
Not specified	509	74.4%	147		
Total	684		203		
Location
Anaesthetic room	17	2.5%	3	17.6%	0.64
Cardiology laboratory	9	1.3%	5	55.6%	2.01
Free-standing day surgery	2	0.3%			
Gastroenterology department	10	1.5%	3	30.0%	1.09
Operating theatre	540	78.9%	149	27.6%	Divisor
Operating theatre reception	3	0.4%			
Post-anaesthesia care unit	38	5.6%	17	44.7%	1.62
Radiology department	16	2.3%	9	56.3%	2.04
Other	29	4.2%	13	44.8%	1.62
Not specified	20	2.9%	4		
Total	684		203		
Anaesthetist grade
Specialist anaesthetist	570	83.3%	178	31.2%	Divisor
Post-fellowship anaesthetist	24	3.5%	6	25.0%	0.80
Trainee	59	8.6%	13	22.0%	0.71
Non-specialist	7	1.0%	1	14.3%	0.46
Other	6	0.9%	3	50.0%	1.60
Not specified	18	2.6%	2		
Total	684		203		
Specialty
Cardiac	33	4.8%	13	39.4%	1.22
Endoscopy	43	6.3%	17	39.5%	1.23
Ear, nose and throat	24	3.5%	5	20.8%	0.65
General	149	21.8%	48	32.2%	Divisor
Maxillofacial	7	1.0%	2	28.6%	0.89
Neurosurgery	23	3.4%	6	26.1%	0.81
Obstetrics and gynaecology	59	8.6%	2	3.4%	0.11
Orthopaedic	153	22.4%	51	33.3%	1.03
Plastics	22	3.2%	6	27.3%	0.85
Procedure not involved	3	0.4%	2	66.7%	2.07
Radiological	13	1.9%	5	38.5%	1.19
Thoracic	14	2.0%	0	0.0%	0.00
Urology	42	6.1%	9	21.4%	0.67
Vascular	68	9.9%	31	45.6%	1.42
Other	30	4.4%	6	20.0%	0.62
Not specified	1	0.1%	0		
Total	684		203		

ASA PS: American Society of Anesthesiologists Physical Status Classification

### Airway, aspiration and other respiratory events

There was a total of 72 cardiac arrests associated with an airway event (*n* = 32), breathing or respiratory difficulty (*n* = 28) or aspiration or regurgitation (*n* = 12) (Table 2). Airway obstruction occurred in 20 cases, with a timing ranging from immediately preoperatively through to the postoperative period. In 15 of these 20 cases, patients were reported to have a BMI greater than 30 kg/m^2^. Sedation along with non-invasive ventilation had been used in seven cases. At induction, there were five cases of difficult or failed intubation that resulted in hypoxia and cardiac arrest. Two of these cases were not anticipated; one was in an obese pregnant patient, and another had unknown subglottic stenosis. Of the three cases involving an anticipated difficult airway, two patients were septicaemic, one from epiglottitis; the third patient was a multitrauma patient. There was one case of failed tracheostomy in a patient whose airway obstructed during an awake tracheostomy insertion attempt after 2 h of trying an awake fibreoptic intubation. Of the 10 airway events which occurred during the maintenance phase, seven arrested due to airway obstruction. One case was due to a mucous plug causing hyperinflation and hypoxic arrest; the second due to a tracheostomy that was facing cephalad through the vocal cords into the oropharynx. There were three cases of laryngospasm on emergence that resulted in cardiac arrest, of which two cases involved anaesthetic trainees. In all three cases positive pressure did not break the laryngospasm. Airway obstruction progressed to a cardiac arrest in five patients on emergence in the operating theatre, three while PACU and one case in ICU.

**Table 2.** Classification of 684 perioperative cardiac arrests identified from the webAIRS database by main category, subcategories, timing and mortality.

	Pre-op	Induction	Maint.	Emergence	PACU	Ward	HDU/ICU	Total	Total %	Death	% of total	Mortality % of row
**Category**												
**Airway events**	**1**	**9**	**10**	**8**	**3**	**0**	**1**	**32**	**4.7%**	**8**	**3.9%**	**25.0%**
Airway obstruction	1	3	7	5	3	0	1	20	2.9%	5	2.5%	25.0%
ETT blocked/kinked	0	0	1	0	0	0	0	1	0.1%			
Intubation difficult	0	3	1	0	0	0	0	4	0.6%	1	0.5%	25.0%
Intubation failed	0	2	1	0	0	0	0	3	0.4%	1	0.5%	33.3%
Laryngospasm	0	0	0	3	0	0	0	3	0.4%			
Tracheostomy (emergency event)	0	1	0	0	0	0	0	1	0.1%	1	0.5%	100.0%
**Anaphylaxis**	**1**	**64**	**22**	**1**	**0**	**0**	**0**	**88**	**12.9%**	**7**	**3.4%**	**8.0%**
Cardiovascular signs	0	43	16	1	0	0	0	60	8.8%	6	3.0%	10.0%
Multiple—CVS, resp. and other	0	18	5	0	0	0	0	23	3.4%	1	0.5%	4.3%
Respiratory signs	1	3	1	0	0	0	0	5	0.7%			
**Aspiration or regurgitation**	**0**	**6**	**2**	**1**	**3**	**0**	**0**	**12**	**1.8%**	**4**	**2.0%**	**33.3%**
Aspiration	0	0	0	0	1	0	0	1	0.1%			
Gastric contents	0	6	2	1	2	0	0	11	1.6%	4	2.0%	36.4%
**Breathing and respiratory**	**0**	**7**	**7**	**7**	**3**	**3**	**1**	**28**	**4.1%**	**7**	**3.4%**	**25.0%**
Bronchospasm/asthma	0	2	0	0	0	0	0	2	0.3%	2	1.0%	100.0%
Circuit disconnection	0	1	1	0	0	0	0	2	0.3%			
Hyperinflation	0	2	1	4	0	0	1	8	1.2%			
Other	0	0	1	0	0	0	0	1	0.1%			
Pneumothorax	0	1	3	0	0	0	0	4	0.6%			
Respiratory arrest (unintended)	0	0	1	3	3	3	0	10	1.5%	5	2.5%	50.0%
Ventilation difficulty/failure	0	1	0	0	0	0	0	1	0.1%			
**Cardiovascular**	**7**	**92**	**257**	**19**	**25**	**14**	**10**	**424**	**62.0%**	**147**	**72.4%**	**34.7%**
Blood loss >patient blood volume	0	0	2	0	0	0	0	2	0.3%	2	1.0%	100.0%
Blood loss—sudden or severe	5	12	56	0	1	1	4	79	11.5%	41	20.2%	51.9%
Bradycardia (abnormal rhythm)	0	4	8	0	2	0	0	14	2.0%	1	0.5%	7.1%
Bradycardia (sinus rhythm)	0	5	14	1	0	0	0	20	2.9%	3	1.5%	15.0%
Cardiac failure	0	0	2	2	1	0	0	5	0.7%	2	1.0%	40.0%
Cardiac tamponade	0	2	6	0	0	1	0	9	1.3%	7	3.4%	77.8%
Dysrhythmia (other)	1	2	8	2	3	2	0	18	2.6%	6	3.0%	33.3%
Embolism (air)	0	0	3	0	1	0	0	4	0.6%	1	0.5%	25.0%
Embolism (venous)	0	3	12	2	1	0	1	19	2.8%	13	6.4%	68.4%
Embolism amniotic	0	0	3	0	0	0	0	3	0.4%			
Embolism BCIS	0	0	21	0	0	0	0	21	3.1%	14	6.9%	66.7%
Embolism CO_2_	0	0	1	0	0	0	0	1	0.1%			
Embolism fat	0	0	7	0	1	0	0	8	1.2%	6	3.0%	75.0%
Hypotension	0	43	13	8	1	1	1	67	9.8%	17	8.4%	25.4%
Myocardial infarction	0	6	12	2	6	7	4	37	5.4%	26	12.8%	70.3%
Myocardial ischaemia	0	1	0	0	1	0	0	2	0.3%	1	0.5%	50.0%
Other	0	0	0	0	1	0	0	1	0.1%	1	0.5%	100.0%
Tachycardia (abnormal rhythm)	0	2	18	1	2	2	0	25	3.7%	5	2.5%	20.0%
Tachycardia (sinus rhythm)	0	0	0	1	0	0	0	1	0.1%			
Vasovagal syndrome	1	12	71	0	4	0	0	88	12.9%	1	0.5%	1.1%
**Medical device or equipment**	**0**	**0**	**3**	**1**	**0**	**0**	**0**	**4**	**0.6%**	**0**	**0.0%**	**0.0%**
Device (malfunction)	0	0	1	0	0	0	0	1	0.1%			
Device (user error)	0	0	2	1	0	0	0	3	0.4%			
**Medication (other than anaphylaxis)**	**0**	**8**	**22**	**5**	**0**	**0**	**0**	**35**	**5.1%**	**3**	**1.5%**	**8.6%**
Untoward reaction (other)	0	7	20	5	0	0	0	32	4.7%	3	1.5%	9.4%
Wrong dose (overdose)	0	1	1	0	0	0	0	2	0.3%			
Wrong dose (underdose)	0	0	1	0	0	0	0	1	0.1%			
**Miscellaneous**	**2**	**14**	**18**	**3**	**4**	**2**	**3**	**46**	**6.7%**	**25**	**12.3%**	**54.3%**
Hyperkalaemia	0	1	7	1	1	0	0	10	1.5%	3	1.5%	30.0%
Hypoglycaemia	0	0	0	0	1	0	0	1	0.1%			
Hypokalaemia	0	1	0	0	0	0	0	1	0.1%			
Severe sepsis	2	12	11	2	2	2	3	34	5.0%	22	10.8%	64.7%
**Neurological**	**1**	**7**	**4**	**0**	**1**	**1**	**1**	**15**	**2.2%**	**2**	**1.0%**	**13.3%**
Convulsions	0	1	0	0	0	0	0	1	0.1%			
High spinal/epidural	0	4	2	0	0	1	1	8	1.2%	1	0.5%	12.5%
LA toxicity	1	2	2	0	1	0	0	6	0.9%	1	0.5%	16.7%
**Total**	**12**	**207**	**345**	**45**	**39**	**20**	**16**	**684**	Arrests	**203**	Deaths	
**Total (%)**	**1.8%**	**30.3%**	**50.4%**	**6.6%**	**5.7%**	**2.9%**	**2.3%**					
**Death (*n*)**	**7**	**42**	**99**	**12**	**18**	**14**	**11**					
**Death (%)**	**3.4%**	**20.7%**	**48.8%**	**5.9%**	**8.9%**	**6.9%**	**5.4%**					
**Mortality rate for the column**	**58.3%**	**20.3%**	**28.7%**	**26.7%**	**46.2%**	**70.0%**	**68.8%**					

*n* = number of cases; % = percentage; Pre-op = pre-operative phase of anaesthesia after admission to the operating theatre and before induction of anaesthesia; Induction = starts when the anaesthetic drugs are given and continues for the first 10 min thereafter; Maint. = during the maintenance phase of anaesthesia; Emerg = the emergence phase of anaesthesia and before admission to PACU; Ward = after discharge from PACU but not to HDU or ICU. Other is a subcategory that either could not be categorised or multiple precipitating events occurred at the same time. Blood loss >patient blood volume = blood loss greater than the patient’s blood volume.

PACU: post-anaesthesia care unit; HDU: high dependency unit; ICU: intensive care unit; ETT: endotracheal tube; CVS: cardiovascular system; Resp.: respiratory system; BCIS: bone cement implantation syndrome; CO_2_: carbon dioxide; LA: local anaesthetic

Of the 12 reports of aspiration or regurgitation, all except one occurred during an emergency procedure. The only elective case was during upper endoscopy for a duodenal stent in a patient with obstructive pathology receiving palliative care. Two regurgitation of gastric contents occurred during maintenance of anaesthesia using first-generation laryngeal masks for emergency cases. One parturient with pregnancy-induced hypertension had an eclamptic seizure, aspirated and arrested. Aspiration during emergence or in the PACU occurred at the end of emergency cases. In each of these reports, the patient transiently lost consciousness and aspirated gastric contents.

There were 28 cases of other breathing or respiratory events that resulted in cardiac arrest. There were two cases of bronchospasm on induction, and two breathing circuit disconnections during induction or maintenance of anaesthesia that were missed. There were eight cases of hyperinflation that resulted from prolonged Valsalva manoeuvres or due to coughing or straining when patients were lightly anaesthetised. There were four cases of pneumothorax, due to jet ventilation (*n* = 2) or surgical error (*n* = 2). One case of hypoxia occurred during one-lung ventilation in which the patient was found to have severe right ventricular failure. Of the 10 cases of respiratory arrest, eight were reported to be secondary to opioid-induced apnoea or partial paralysis. There was a single case of ventilatory failure due to an unrecognised pleural effusion, which was found after return of spontaneous circulation (ROSC); 2 l of fluid were immediately drained. A decrease in air entry had been observed preoperatively.

### Anaphylaxis

There were 88 cases of anaphylaxis that led to cardiac arrest. Cardiovascular signs were reported in 60 cases, respiratory signs in five cases and multiple signs in 23 cases. Common suspected allergens included antibiotics, muscle relaxants and chlorhexidine, including chlorhexidine-impregnated central venous catheters. Others included patent blue dye, intravenous contrast agents, opioids and one related to sugammadex. One case failed to respond to intravenous fluids or drug therapy and was successfully placed on cardiopulmonary bypass (CPB). After stabilisation they were weaned from CPB and transferred to the ICU. Three other reported cases of anaphylaxis and cardiac arrest were managed with extracorporeal membrane oxygenation (ECMO).

### Cardiovascular

There were 424 cardiac arrests due to a cardiovascular cause. Eighty-one cardiac arrests were due to blood loss. The most common procedures involved either the abdominal aorta, or the iliac or femoral arteries (*n* = 22). There were 14 cases of cardiac arrest during a laparotomy and 14 with upper gastrointestinal bleeding. Three reporters mentioned a lack of a preoperative blood group and hold, or a delay in receiving blood products.

Reflex vagal arrhythmias resulting in a prolonged sinus pause with loss of output were reported in 88 reports. Common precipitating causes included pneumoperitoneum (*n* = 23), retraction/manipulation of intra-abdominal organs/peritoneum (*n* = 18), airway manipulation (*n* = 8), genitourinary procedures (*n* = 7), during colonoscopy (*n* = 4), vagal response to painful stimuli (*n* = 4) and manipulation of the eye (*n* = 3). Of 88 cases of cardiac arrest associated with a vasovagal reflex, 66 required CPR. Twenty-two patients were reported to have prolonged asystole with a loss of output. Of these, 15 responded to atropine or glycopyrrolate, three responded to adrenaline, while four met the study criteria for cardiac arrest, but native rhythm returned once vagal stimulation was ceased. These latter cases were included as the reporter coded them as cardiac arrests.

Embolism was reported in the narrative of 56 reports, including 21 cases of bone cement implantation syndrome, 19 venous thromboembolic events, eight fat emboli, four air emboli, three amniotic fluid emboli and one carbon dioxide embolus. In each incident, there was a sudden drop in blood pressure, and in six cases a drop in end-tidal carbon dioxide. One patient was placed on CPB after the arrest. The use of transthoracic or transoesophageal echocardiography during the arrest to confirm diagnosis and guide management was mentioned in 17 cases.

There were 39 cases of myocardial ischaemia or infarction. Eighteen of these were emergency cases, of which 14 died. Nineteen arrests were reported during induction or maintenance. In 14 cases the reporter mentioned significant pre-existing cardiac disease. In the postoperative period, patients were commonly found to have a high-grade coronary stenosis that was not recognised preoperatively. A mechanical chest compression device was used in three patients who arrested in a coronary angiogram suite. None of these patients survived.

Cardiac tamponade occurred in nine cases, of which seven were iatrogenic surgical complications during interventional endovascular procedures. The remaining two were due to a pre-existing tamponade awaiting surgical drainage. Cardiac failure led to perioperative cardiac arrest in five cases.

Pulmonary hypertension was highlighted by reporters as a significant risk factor in 32 (4.7%) reports.

Hypotension was noted in 67 reports. The majority of the 43 patients who arrested on induction due to hypotension were ASA PS 3 to 5, or older than 70 years. Time pressure was reported to be an important contributor in three incidents that occurred during induction. Of the 13 cases that arrested during the maintenance of anaesthesia, five occurred during a change of patient’s position (i.e. supine to prone or lateral). Reporters commonly stated that these patients were hypovolaemic, which was assessed either clinically or via echocardiography. Of the eight cases of hypotension on emergence, four patients arrested when they were transferred from the operating table to their beds. The three cases that occurred in the postoperative period were all in patients over the age of 70 years and occurred in PACU, ICU or on the ward.

Arrhythmias were associated with 78 cases of perioperative cardiac arrest. There were 34 reports of bradycardia, including two cases during electroconvulsive therapy (ECT), four immediately post cardioversion and in one case due to the Cushing’s reflex from increased intracranial pressure. All of these cases either received CPR or were coded as a cardiac arrest by the reporter. There were 18 cases of dysrhythmia, including one case during ECT and another due to inappropriate discharge of a spinal cord stimulator while being inserted. There were 26 cases of perioperative cardiac arrest associated with tachyarrhythmias.

### Medical devices and equipment

There were four perioperative cardiac arrests associated with malfunction of a medical device or medical equipment. Two cases involved occlusion of the anaesthetic circuit causing hyperinflation of the lungs and cardiac arrest. This included one case where the surgical equipment rolled over the expiratory breathing limb and occluded the anaesthetic circuit, the second was due to a faulty positive end-expiratory pressure valve. In another case, an undetected endotracheal cuff leak in a morbidly obese patient resulted in inadequate ventilation that progressed to a hypoxic cardiac arrest. A mis-deployed aortic valve during transcatheter aortic valve implantation caused torrential aortic regurgitation and cardiac arrest. After ROSC was achieved, a second valve was deployed within the first valve.

### Medication

Medication issues were associated with 35 cardiac arrests. Inadvertent boluses of medication that caused profound hypotension and cardiac arrest included vancomycin, nitrates, beta-blockers, prostacyclin, patent blue dye and protamine. There were two cases of inadvertent intravenous administration of adrenaline. There were asystolic cardiac arrests after the administration of sugammadex (*n* = 5), remifentanil (*n* = 7) and repeated doses of suxamethonium (*n* = 3). There was one case in which a vasopressor infusion was disconnected, and this was not recognised prior to the arrest.

### Miscellaneous

There were 46 cases that were classified using the main category ‘miscellaneous’. These included 10 cases of hyperkalaemic cardiac arrest. Three of these occurred during reperfusion after prolonged tourniquet use in lower limb orthopaedic procedures (two more than 2 h and one more than 3 h). Two happened after reperfusion during liver transplant, two in patients with end-stage renal failure, and one after surgical release for compartment syndrome. In two reports, there was no apparent cause. One arrest occurred on induction in a patient with hypokalaemia but with no other identified cause.

There was one case of hypoglycaemia that the reporter thought was due to prolonged fasting leading to a low blood sugar level (1.6 mmol/l), which was measured during the arrest. Thirty-four cases were associated with severe sepsis. Of these, 31 were emergency cases, 15 were performed out-of-hours and 22 died. Nineteen cases were undergoing general surgery, which included one patient who developed intra-abdominal sepsis after a procedure for an infected femoral nail. Of five gastroscopy cases, four were in the setting of endoscopic retrograde cholangiopancreatography for gallstone pancreatitis.

### Neurological

There were 15 cases of cardiac arrest following neurological events. Eight followed an inadvertent ‘total spinal’ during a neuraxial technique. In three cases an epidural was topped up resulting in a high block. In two of these three cases the reporter suspected an inadvertent intrathecal catheter. One reporter tested the fluid that was aspirated and confirmed the intrathecal location. One arrest occurred during a surgical epidural injection of local anaesthetic during spinal surgery. There were three cases where the use of a combined spinal/epidural (CSE) resulted in a total spinal despite a standard dose of local anaesthetic being administered in each case; it was unclear whether the epidural was loaded after the CSE was placed.

There were six cases of local anaesthetic toxicity that resulted in cardiac arrest. Four cases occurred during a regional or neuraxial technique in which the syringe was aspirated prior to injection of local anaesthetic but immediate cardiovascular collapse resulted. There were two cases in which local anaesthetic was injected intravenously, which was subsequently thought to be due to a possible medication error.

### Post-crisis management of cardiac arrest and outcome

Of 684 patients who had a cardiac arrest, 166 (24.3%) patients were resuscitated and discharged to the ward, 315 (46.1%) were transferred to an ICU and 203 (29.7%) died. Of the 106 patients who had a cardiac arrest out of hours, 88 were emergency cases and 47 patients died in hospital.

The number of deaths and the mortality rate for each of the main categories is shown in the right-hand column of Table 2. The greatest number of deaths (*n* = 147) was following a cardiovascular precipitant, which included 72% of all deaths and a mortality rate of 35% from 424 arrests. This was followed by miscellaneous, with 12% of all deaths and a mortality rate of 54%. The mortality rate for some of the subcategories was higher but the numbers were low. These included emergency tracheostomy (*n* = 1, 100% mortality), bronchospasm (*n* = 2, 100% mortality) and blood loss greater than the patient’s blood volume (*n* = 2, 100% mortality). Airway events overall had a mortality rate of 25%, anaphylaxis (8%), aspiration or regurgitation (33%), medication (8.6%) and neurological (13.3%). The other subcategories with a mortality rate of 50% or more included respiratory arrest (50%), blood loss sudden or severe (53%), cardiac tamponade (78%), several categories of embolism (around 70–75%), myocardial infarction (70%), myocardial ischaemia (50%) and 34 cases of severe sepsis with a mortality of 65% (*n* = 22).

The authors considered that 183 of the 684 cardiac arrests were likely to be attributable to anaesthesia factors alone, and a further 93 to surgical factors alone. The remaining 408 cases were considered to have a multifactorial aetiology, with a combination of anaesthetic, surgical and complex patient factors. In each case, attributing cause did not necessarily imply error, as many of the factors might have been outside the control of either the anaesthetist or the surgeon.

## Discussion

In this overview we have presented 684 cases of cardiac arrest reported to webAIRS between September 2009 and March 2022 with detailed description of the demographic and procedural factors involved, and a detailed analysis of the site and timing of the arrests and their outcomes, including deaths. While it is not possible to determine either the incidence or the factors associated with cardiac arrest from any de-identified voluntary reporting system, the large quantity of data collected provides an insight into the types of situations in which cardiac arrest occurs, and the outcomes in these various situations.

Many findings are unsurprising, with the majority of reported incidents involving patients of age greater than 60 years and an ASA PS of 3 to 5, undergoing emergency procedures and more major procedures. The most common precipitant being cardiovascular was also not surprising, with anaphylaxis, medication issues and airway events being other common precipitants. Although general surgery and orthopaedic surgery had the highest proportion of arrests in this dataset, this may reflect the greater number of cases performed by these surgical specialties in Australia.^9^

Apart from contributory or precipitating factors, the data provide additional information on outcomes following cardiac arrest, with cardiac arrest associated with miscellaneous precipitants (mostly accounted for by severe sepsis) had the highest mortality rate of 54% and accounting for 25% of the deaths overall. This was followed by cardiovascular precipitants with 147 of 424 dying (35%) but accounting for over 72% of the deaths overall, airway or breathing events (25%, 3.9% overall), and anaphylaxis (8%, 3.4% overall). Pulmonary hypertension was often reported as an important contributing comorbidity in the narrative analysis by reporters. Pulmonary hypertension is a known significant risk factor for cardiac arrest, and is known to be associated with worse outcomes.^10^ While risk factors such as such as cancer, hepatic disease or obesity have a weak association with mortality and cardiac arrest, these factors were not identified from the narrative reports included in this study.^2^ These findings highlight that perioperative risk discussion is essential for any high-risk patient, including those of older age, with a higher ASA PS or with increased frailty, despite routine suspension of advanced health directives or resuscitation orders over the perioperative period.^11,12^

**Figure 2.**
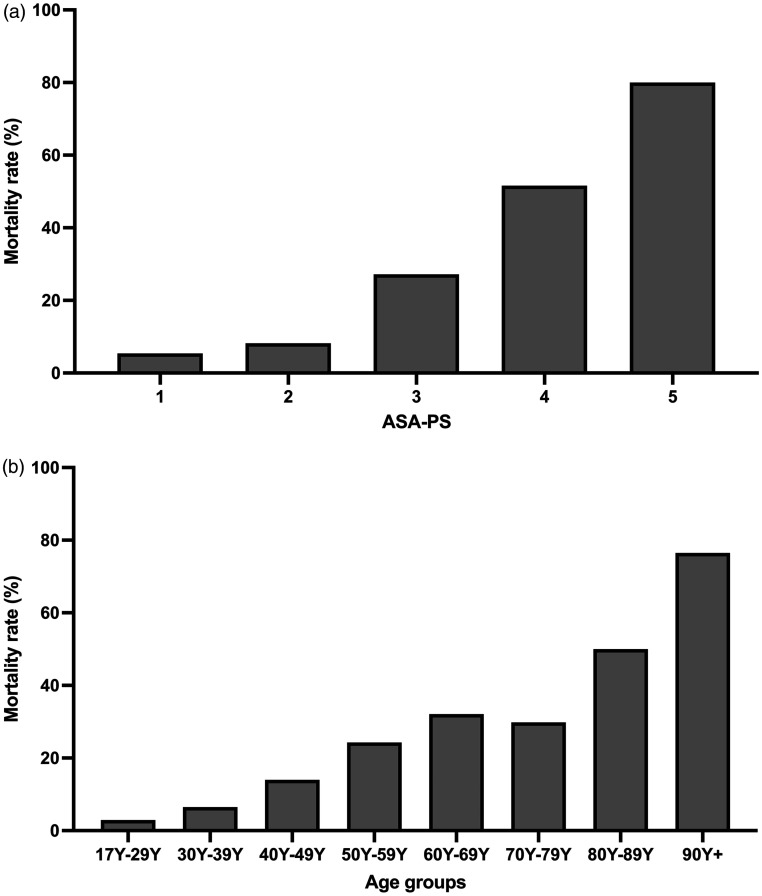
Mortality associated with 684 perioperative cardiac arrests identified from the webAIRS database. (a) Mortality represented as a percentage within each American Society of Anesthesiologists Physical Status Classification (ASA PS); (b) mortality represented as a percentage within each age group (in years (Y)).

Mortality measured as a percentage of arrests was higher with increasing urgency of surgery, particularly out of normal working hours (Table 1), increasing age and ASA PS (Figure 2). These findings likely reflect that an increased case acuity in elderly and sicker patients at a time of reduced workforce and possible fatigue of the theatre team might contribute to these findings. These findings were again confirmed when the risk ratio of mortality after a cardiac arrest is calculated. While the relative risks described above are based on several assumptions, the database provides unique and compelling information about the influence of patient and procedural factors on the outcome of cardiac arrest, should a cardiac arrest occur. For example, the risk of death if a cardiac arrest occurs in an ASA PS 4 patient is over nine times the risk of if it occurs in an ASA PS 1 patient (Table 1). For ASA PS 5 patients this is nearly 15 times higher. Males suffering a cardiac arrest had a 44% higher risk of death than females. Urgent procedures were associated with a 2.7 relative risk of death in the event of a cardiac arrest versus elective procedures, while out-of-hours procedures were associated with a 56% higher risk of death up until 22:00 h, and an over 70% higher risk of death overnight if cardiac arrest occurred. Vascular, cardiac, radiological and endoscopy procedures were associated with the highest risk of death if a cardiac arrest occurred, while the relative risk of death following a cardiac arrest in obstetric/gynaecological patients was very low (0.1 *vs*. general surgical procedures) (Table 1). It is important to note that many of these risk factors may not have been independent. The risk of death following a cardiac arrest in patients managed by non-specialist anaesthetists was also lower than for those managed by specialist anaesthetists, again possibly related to the acuity of the patients involved.

Most cardiac arrests occurred during the induction (30.3%) or maintenance phases (50.4%) of anaesthesia (Table 2). Similarly, most deaths following cardiac arrest occurred during these phases; 21% were associated with induction (*n* = 42) whereas almost half were associated with maintenance (*n* = 99, 49%). However, when viewed as the number of deaths as a proportion of the arrests for each perioperative phase, induction (20%), maintenance (29%) and emergence (27%) had the best outcomes.

There were fewer arrests reported during emergence (*n* = 45, 6.6%), in PACU (*n* = 39, 5.7%), in the ward (*n* = 20, 2.9%) or in ICU or HDU (*n* = 16, 2.3%). However, the death rate was higher in PACU (46%), the ward (70%) and in ICU or HDU (69%). It was also higher in the preoperative phase (58%) compared with an overall mortality rate of 30%. The rate was slightly lower than 30% in the cases during induction (21%), maintenance (29%) and emergence (27%). The reason for this is not known, but in the operating theatre there is a high level of monitoring, and an anaesthetist is always present at the monitoring station. In the other areas there are variable levels of staff to patient ratios and monitoring used. In the ward the reports included respiratory arrest (*n* = 3), myocardial infarction (*n* = 7), tachycardia (*n* = 2), other dysrhythmia (*n* = 2), severe sepsis (*n* = 2), cardiac tamponade (*n* = 1), hypotension (*n* = 1), blood loss (*n* = 1) and high spinal/epidural block (*n* = 1), with 14 deaths from the 20 cases (70% mortality). There was insufficient detail to know whether these cases represented a failure to rescue or whether inadequate monitoring played a part, but the severity of the precipitating events suggests that these cases might have benefitted from monitoring in a 24-h postoperative care ward or in HDU, rather than a regular surgical ward.^13^ The high death rate in ICU and HDU included predominantly cardiovascular events (*n* = 10) and severe sepsis (*n* = 3) among the 16 cases and reflects the higher postoperative risk and therefore the reason for the admission to ICU or HDU.

Of the 203 deaths, the majority were in the cardiovascular main category (*n* = 147) with a mortality rate of 35%. However, when looking at the subcategory groups, the highest mortality (100%) after cardiac arrest was emergency tracheostomy (*n* = 1), bronchospasm/asthma (*n* = 2), blood loss greater than the patient’s blood volume (*n* = 2) and other (*n* = 1). The last case was coded as other but was an elderly patient having a femoral nail who had cardiovascular decompensation at the end of the case and died without an obvious diagnosis.

Apart from the summary data, further details of individual cases within the various precipitant categories are presented in the Results section, to provide scope for further scrutiny within these categories. These details provide a rich basis for the analysis of human and system factors related to these incidents. However, their heterogeneity is too broad to be covered in an overview of this type, other than confirmation that a range of factors is typically involved, often including a combination of patient, human, task and system factors. Analyses of these factors and further details have been included in many previous webAIRS publications and reports of specific incident categories. Detailed subcategory analysis of incidents causing cardiac arrest, peri-arrest or death will be included in future specific category reports.

While the authors considered that 183 of the 684 cardiac arrests were related to anaesthesia factors alone, this does not necessarily imply that they considered a human error had occurred. It is possible that cardiac arrest could not be avoided, despite optimum choice and conduct of a particular anaesthetic technique. Nevertheless, each case would require scrutiny to determine whether the arrest could have been prevented or better managed, with learning points applied at a local and a national level. The same would apply to arrests considered to be related to surgical factors alone, and to those considered to be related to a combination of factors.

The first event in the chain of events could be regarded as a precipitating event or, alternatively, the first indication that a critical incident is occurring. In Table 2, the main categories for these events show ‘Airway’ as precipitating 4.7% of the cardiac arrests, ‘Anaphylaxis’ (12.9%), ‘Breathing or respiration’ (4.1%), ‘Cardiovascular’ (62%), ‘Drugs’ (medications 5.1%) and ‘Other’ (11%). The irregular capitalisation in the previous sentence shows a close similarity to the ‘ABC’ component of the aide memoire ‘Drs ABCD’ for the diagnosis and management of critical incidents. An alternative is ‘Circulation Airway Breathing (CAB)’, which has been shown in a double-blind trial to be superior to ‘ABC’.^14^ This would be in keeping with the findings in this study as cardiovascular was the most frequent precipitating event (62%). There have been other extensions such as ‘ABCDE’ but in the latter example ‘D’ stands for Disability and ‘E’ for Exposure.^15^ The authors suggest that if the aide memoire was adapted for anaesthesia, ‘D’ could stand for Drugs and ‘E’ for Exceptions. The latter, ‘E’, would include everything not included in ‘ABCD’.

The subcategories of these initial events were analysed and are listed in Table 2. It was found that the frequency of the subcategory of the events reports depended on the timing of the events. In the pre-induction phase, there were 12 incidents which accounted for 1.8% of all the arrests, with five related to blood loss, followed by two related to severe sepsis and one each for airway obstruction, anaphylaxis, arrhythmia, vasovagal and local anaesthetic toxicity. In contrast, the most frequent subcategories in the induction phase were anaphylaxis (*n* = 64), unexplained hypotension (*n* = 43), blood loss (*n* = 12), vasovagal (*n* = 12) and severe sepsis (*n* = 12). There was a wide range of other events, as shown in Table 2. One of the management difficulties is knowing whether hypotension causing cardiac arrest at induction is due to anaphylaxis, a relative overdose of induction drugs, hypovolaemia, cardiac pathology or other patient factors. Suspected anaphylaxis was observed in 64 cases during induction compared with 24 in the other phases of perioperative care. Additionally, there may be other signs such as respiratory (*n* = 3), a rash (*n* = 0) or multiple signs (*n* = 18), but the majority in this study were cardiovascular alone (*n* = 43). Coincidentally, there were 43 cases of unexplained hypotension that did not appear to be due to anaphylaxis during induction. It was assumed that these cases were related to a combination of anaesthetic induction drugs, patient comorbidities or hypovolaemia. It was interesting to note that there were no cases in this series where hypertension was followed by cardiac arrest. The remainder of the webAIRS database was searched, and there were 53 cases during the same reporting period as this cardiac arrest study and two died (3.8%). None of these cases was eligible for this cardiac arrest study. Whereas there were 380 cases of hypotension in the same period in the whole webAIRS database and 34 died (8.9%), 67 cases were examined in our cardiac arrest analysis and of these 17 (25.4%) died. These results might reflect a pendulum swing whereby avoiding hypertension during anaesthesia, and particularly during induction, might have the unintended consequence of hypotension, which has a higher death rate in the webAIRS database as well as in the cardiac arrest study.

During maintenance a different pattern was observed. The predominant cardiovascular subcategories (*n* = 257) were blood loss (*n* = 58), vasovagal (*n* = 71), other arrhythmias (*n* = 48), embolism (*n* = 47), hypotension (*n* = 13), myocardial infarction (*n* = 12), cardiac tamponade (*n* = 6) and cardiac failure (*n* = 2). Blood loss, vasovagal episodes and arrhythmias are likely to be easily diagnosed with routine monitoring and observation. Unexplained hypotension, myocardial infarction, embolism, tamponade and cardiac failure may be more difficult to diagnose and other investigations such as echocardiography might be useful. Anaphylaxis (*n* = 22) was less common than during induction and might follow the administration of a single drug during maintenance, making the diagnosis potentially easier. Airway events (*n* = 10) were slightly higher than during induction (*n* = 9) and included airway obstruction (*n* = 7), a blocked endotracheal tube, a difficult intubation and a case of failed intubation. There were two cases of regurgitation of gastric contents during maintenance. Breathing and respiratory accounted for seven cases, which included pneumothorax (*n* = 3), a circuit disconnection, a hyperinflation episode, a respiratory arrest and another, unspecified, respiratory problem. Equipment errors were followed by cardiac arrest in three cases, medication errors other than anaphylaxis in 22 cases, 20 of which were untoward reactions, as well as one wrong dose and one wrong drug. In the miscellaneous category (*n* = 18), seven were associated with hyperkalaemia and 11 with severe sepsis. The neurological category included two high spinal or epidural blocks and two cases of local anaesthetic toxicity. There were four cases of anaphylaxis refractory to almost all the measures in the Australian and New Zealand Anaesthetic Allergy Group^16^ guidelines for refractory management of anaphylaxis which did respond to CPB (*n* = 1) or ECMO (*n* = 3) as per the final measure specified in the guidelines. ECMO was also considered or used in several other patients with arrests from different aetiologies.

Since the AIMS report in 1993, there have been few studies reporting perioperative cardiac arrest data in Australia and New Zealand. Recently, however, the Audit of Airway incidents during Anaesthesia (AAA) report included data on cardiac arrests due to airway management.^17^ This report had stricter reporting criteria but was from a small subset of hospitals. Nevertheless, in terms of airway incidents progressing to cardiac arrest, the findings were similar. For example, ASA PS 1 was associated with the fewest cardiac arrests and deaths, while there were similar percentages of arrests and deaths between the findings of this analysis and the AAA report.^17^ Similarly, patient sex did not influence the rate of cardiac arrests, while age above 60 years was associated with a higher rate. Equally, emergency cases were associated with a higher rate of cardiac arrests and death if cardiac arrest occurred, particularly if out of hours. A strength of the AAA study was the inclusion of denominator data. The similarity in the demographic and procedural influences in the AAA report to the factors identified in this webAIRS report increases the face validity of these webAIRS findings.

More recently, the American National Anesthesia Clinical Outcomes Registry (NACOR) study of perioperative cardiac arrests reported a mortality rate of 58.4%.^18^ This was one of the largest studies assessing intraoperative and immediately postoperative cardiac arrest. The authors analysed 1,691,472 cases from across 49 facilities, 27 practices and 408 providers from 2010 to 2013. A total of 951 cardiac arrests were identified (5.6 per 10,000 cases). There were 396 survivors and 555 deaths.^18^ The large data set from the NACOR study was based upon hospital coding with few descriptors to determine underlying causes of arrest. From several smaller studies, risk factors for perioperative cardiac arrest included patient factors (age over 50 years or paediatric, ASA PS classification of 3 or more and male gender, surgical factors (emergency cases and high-risk abdominal and thoracic procedures) and anaesthetic factors (general anaesthesia and out-of-hours procedures having a higher risk).^18–22^ A recent systematic review that examined 87 articles and over 21 million surgeries between 1955 and 2008 found that there has been a significant decline in anaesthesia mortality and cardiac arrest in high-income countries, despite an increased number of older patients presenting with more comorbidities.^23^ It is likely that both clinical and non-clinical interventions including staff education, improved clinical practice and airway management algorithms, and improved monitoring including videolaryngoscopy have undoubtedly decreased the incidence of perioperative cardiac arrest.^4^ However, our findings indicate that there is further room for improvement in terms of both preventing perioperative cardiac arrest and improving outcomes following perioperative cardiac arrest.

It was pleasing to find that most cardiac arrests had been attended by specialist anaesthetists. The findings of this review are also reminders for clinicians to have increased awareness for any changes that can occur when positioning the patient after induction of anaesthesia (i.e. lateral/prone positioning) or prior to extubation when rolling the patient and moving them back to the ward bed. During these periods of movement, any fluid shifts and reductions in preload may be exacerbated by the vasodilatory state secondary to anaesthesia. In addition, it is recommended to have increased vigilance during out-of-hours cases or lengthy procedures where the finish time may be late; the team may be fatigued and limited resources are available.^24^

One purpose of clinical incident reporting, whether at a departmental, hospital, state, national or international level, is to share experiences of near misses, crisis management and unexpected adverse outcomes. The sharing of information reinforces to clinicians that they are not alone in encountering such incidents, no matter how rare or severe they might be. The details of individual incidents may also assist in identifying human or system factors that contributed to the incident or suggest strategies by which similar incidents could be prevented or better handled in future. Perioperative cardiac arrest is fortunately rare. However, this means that only a large database can provide an overview of likely causes and outcomes. This analysis of 684 perioperative cardiac arrests from the webAIRS database represents one of the largest such series ever reported. As webAIRS collects only voluntary, de-identified incidents reports, it is not possible to obtain a numerator for the incidents occurring, either overall or for any particular category. Similarly, denominators for patient demographics or procedures are not readily available.

It is important here to emphasise that these data relate to cardiac arrests reported to webAIRS, which is likely to be only a proportion of total cardiac arrests that occurred during that period. Nevertheless, the large number of reports includes a wide range of cases, precipitants, demographics and outcomes, many of which would resonate with clinicians, indicating that these clinicians are not alone in experiencing such incidents and outcomes, which is a beneficial aspect of incident reporting. Many clinicians may find the summary descriptions of reported incidents within the various categories informative. The data also provide rich scope for further research into further initiatives to prevent cardiac arrests in the perioperative period and to improve outcomes should a cardiac arrest occur.
